# Development, Validation and Application of the Dried Blood Spot Analysis Method for the Determination of Ustekinumab in Patients with Inflammatory Bowel Disease

**DOI:** 10.3390/ph18091253

**Published:** 2025-08-24

**Authors:** Panagiotis-Dimitrios Mingas, Jurij Aguiar Zdovc, Iztok Grabnar, David Drobne, Tomaž Vovk

**Affiliations:** 1 Faculty of Pharmacy, University of Ljubljana, 1000 Ljubljana, Slovenia; panagiotis-dimitrios.mingas@ffa.uni-lj.si (P.-D.M.); jurij@zdovc.net (J.A.Z.); iztok.grabnar@ffa.uni-lj.si (I.G.); 2 Department of Gastroenterology, University Medical Centre Ljubljana, 1000 Ljubljana, Slovenia; david.drobne@kclj.si; 3 Department of Internal Medicine, Faculty of Medicine, University of Ljubljana, 1000 Ljubljana, Slovenia

**Keywords:** ustekinumab, dried blood spots, venous blood, capillary blood, validation, inflammatory bowel disease, ELISA

## Abstract

**Background**: Ustekinumab (UST) is a monoclonal antibody (mAb) used in the treatment of inflammatory bowel disease (IBD). Elevated serum concentrations are typically associated with improved therapeutic outcomes, and therapeutic drug monitoring (TDM) is a useful tool for guiding mAbs treatment. This study aimed to develop a dried blood spot (DBS) method for TDM of UST in patients with IBD. **Methods**: The commercial enzyme-linked immunosorbent assay for plasma samples was optimized for DBS samples and subsequently validated according to international guidelines for classical and DBS-specific validation parameters. It was then applied to analyze serum and DBS samples obtained from venous and capillary blood of IBD patients undergoing UST therapy. **Results**: The method was linear (3–12 mg/L) with acceptable inter-day accuracy (90.1–106%) and precision (<12%). We confirmed that there was no hematocrit effect and that DBS samples were stable for one month under room conditions. A linear model was developed between venous DBS and serum UST concentrations, which showed no systemic bias, and 71% of the samples were within ±20% of the mean. In addition, a linear correlation between venous DBS and capillary DBS samples was established, showing no significant bias, with 84% of samples within ±20% of the mean. Finally, a novel strategy was developed to overcome the limitations of poor-quality samples (irregular shapes) based on area image analysis. **Conclusions**: The newly developed DBS method is the first to enable reliable measurement of UST in capillary blood, appropriate clinical interpretation of the measured concentrations, and remote monitoring of patients in the early phase of therapy.

## 1. Introduction

Ustekinumab (UST) is a human immunoglobulin G1 kappa, monoclonal antibody (mAb). It specifically binds to the p40 subunit, which is common for interleukin (IL)-12 and IL-23, cytokines implicated in natural inflammatory and immune responses [1,2]. UST is indicated for the treatment of adult patients with psoriatic arthritis, moderate-to-severe plaque psoriasis, moderate-to-severe active Crohn’s disease (CD), and moderate-to-severe active ulcerative colitis (UC) [3]. CD and UC are the most prevalent forms of inflammatory bowel disease (IBD), affecting 1% of the population [4]. The pharmacokinetics (PKs) of UST are comparable in patients with UC and CD. The serum concentrations of therapeutic mAbs are dose-dependent and are not significantly influenced by prior treatment with biologics or concurrent immunomodulator therapies, although immunogenicity and disease-specific factors may influence PKs in certain cases [5]. In clinical practice, higher UST serum concentrations are associated with improved clinical and histological outcomes. In particular, studies have shown that UST concentrations ≥ 4.2 mg/L at week 8 are related to a 50% decrease in fecal calprotectin (FC), a relevant biomarker of mucosal healing [6,7,8]. Additionally, studies have demonstrated that measuring UST serum concentrations 1 h after intravenous infusion (UST ≥ 104 mg/L) or during the first two weeks of treatment (UST ≥ 27 mg/L) is associated with biochemical or endoscopic remission [9].

In general, patients’ responses to therapy can differ, and in the case of mAbs, several studies have indicated that the higher the serum concentration of a biologic drug, the higher the chance of beneficial therapeutic outcomes. Therapeutic drug monitoring (TDM) can be a useful tool in decision-making for possible dose adjustments or, if considered necessary, switching to an alternative therapy [10,11]. TDM of mAbs is routinely performed by collecting venous blood samples from patients via venipuncture. However, patients could utilize an alternative method of home sampling without the need to visit specific institutions. This method uses dried blood spots (DBSs), which can be obtained by finger pricking. It is a method characterized by simplicity, and patients can perform it themselves after receiving comprehensible instructions. After the DBS card is spotted with capillary blood, it is air-dried and then mailed to the corresponding laboratory by postal service, where the DBS is extracted. It has already been demonstrated that the concentration of various mAbs can be accurately measured in DBS samples [12,13,14,15,16,17,18,19]. Additionally, the DBS method could serve as a way of reactive TDM; this means that the results of the measurements would be available during the next visit of the patient at the clinic, and the dose of UST could be further adjusted to optimize treatment accordingly [17].

This study aimed to develop a DBS method for the TDM of UST in patients with IBD. We aimed to define the correlation between (venous and capillary) blood and serum UST concentrations. This will enable the clinical interpretation of the determined UST concentration in the blood based on its established therapeutic range in the serum. Therefore, the developed method could serve as an alternative to venipuncture for a specific group of patients.

## 2. Results

### 2.1. Method Validation

#### 2.1.1. Selectivity, Recovery, and Dilution Integrity

For selectivity, drug-free venous blood obtained from five donors was used to prepare blank DBS samples with specified hematocrit (HCT) values (0.2, 0.3, 0.4, 0.5, and 0.6). After the analysis, the responses in the blanks were not significantly different between groups with specific HCT, except for the responses of HCT groups 0.2 and 0.6 (Supplementary Material, Table S1). Since the population studied had a HCT value between 0.3 and 0.5, no corrections were made to the responses of the DBS samples obtained from the patients. The mean recovery for two quality control (QC) concentrations, QC low (QC_L_) and QC high (QC_H_)_,_ at two HCT levels was 90 ± 7% (HCT 0.25) and 82 ± 13% (HCT 0.55), respectively. Dilution integrity was evaluated at two concentrations: 30 and 120 mg/L. The accuracy was 96.0% and 94.5% while precision was 8.3% and 7.7%, respectively, confirming that method can be used up to 120 mg/L (Supplementary Material, Table S2).

#### 2.1.2. Calibration Model, Accuracy, Precision, and Limits of Quantification

The developed method is linear from 3 to 12 mg/L and was evaluated by back-calculating the concentrations of the calibrators. The back-calculated concentrations were within the limits described by the guidelines [20], ≤15% of the nominal value and ≤20% of the lower limit of quantification (LLOQ). The slope and intercept values for the calibration curves are listed in Table 1. The results of precision and accuracy studies are presented in Table 2. The values presented were within the recommended limits of 20% for the LLOQ and ≤15% for the other QC levels. The intra- and inter-day precision of UST was below 13.4% and 11.2%, respectively. The intra- and inter-day accuracy were between 84.2–101.1% and 90.1–106.4%, in that order. The LLOQ was 3 mg/L.

#### 2.1.3. Hematocrit Effect

The results of the effect of HCT on UST in the DBS-QC samples are shown in Table 3. The deviations of the UST concentration compared to the concentration at HCT 0.4 were within 15%. The results of the one-way ANOVA showed that the concentrations at low (*p* = 0.136) and high (*p* = 0.071) QC levels were not influenced by HCT.

#### 2.1.4. Stability

**Figure 1:**
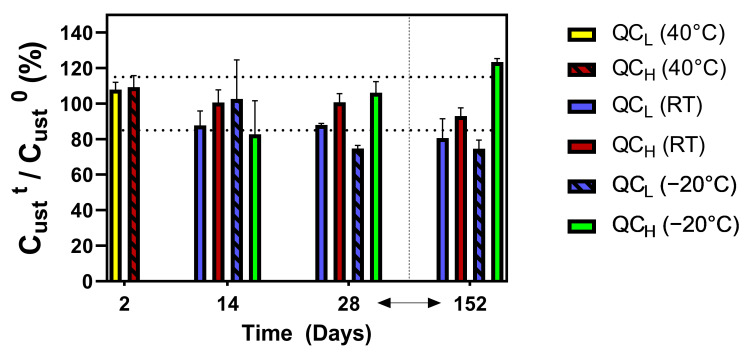
Stability of UST DBS under three different conditions (40 °C, RT, and −20 °C) and two concentrations (QC_L_ and QC_H_). The dotted lines represent a 15% deviation.

The stability of the samples was assessed by spotting two concentrations of UST (QC_L_ and QC_H_) on DBS paper in triplicate, and the cards were stored in airtight bags at room temperature (RT) and in a freezer at −20 °C for up to 5 months. Additionally, DBS cards were stored at 40 °C for 2 days. The results of the stability studies are shown in Figure 1. The samples were stable for one month, except for the QC_L_ at −20 °C, where a decrease of approximately 25.1% was observed. Deviations from the criteria limits were observed for all samples after 5 months of storage, except for the QC_H_ samples stored at RT.

### 2.2. Clinical Validation

#### 2.2.1. Patient Characteristics

Table 4 shows the characteristics of the patients with IBD included in this study population. We included five men and five women with a median age of 52 years; six were diagnosed with UC and four with CD. At the initiation of UST therapy, the median C-reactive protein (CRP) concentration in the patients was 9.5 mg/L, but in six patients, the concentration was below 5 mg/L (normal values), indicating elevated concentrations in four patients. Similarly, the second biochemical marker for IBD, FC, was elevated (>100 mg/kg) in five patients, with values >500 mg/kg in three patients and a median value of 130 mg/kg in the remaining patients. The median induction dose of UST was 390 mg, and all patients were treated with a maintenance dose of 90 mg every 8 weeks, with the exception of one patient who intensified therapy to 90 mg every 4 weeks after 8 weeks of the standard dosing regimen. During therapy, we collected 35 venous and 25 capillary blood samples.

#### 2.2.2. Correlation Between Serum and Dried Blood Spot Venous Ustekinumab Concentrations

The results of the analysis of the relationship between UST concentrations in serum and venous DBSs are shown in Table 5. Three models were evaluated to establish this relationship. Deming regression analysis showed that the slope and intercept for the proportional and linear models (Models 1 and 2) were not significantly different from 1 and 0, respectively, indicating good agreement. Similarly, the Bland–Altman analysis showed no significant mean absolute bias for Models 1 and 2, with 95% limits of agreement (LoA) ranging from −11.0 to 9.9 mg/L for Model 1 and −10.4 to 10.4 mg/L for Model 2, respectively. According to the European Medicines Agency (EMA) acceptance criteria, 60% of the samples predicted by Model 1 and 71% of the samples predicted by Model 2 were within 20% of the mean. Both models met the predefined thresholds for predictive performance, with acceptable mean predictive percent error (MPPE) and mean absolute percentage error (MAPE) values.

In contrast, Model 3, which incorporates HCT to adjust the relationship between serum and DBS concentrations, did not improve the predictive performance. This model exhibited a significant systemic bias, with much wider LoA (−31.1 to 76.8 mg/L) than Models 1 and 2. Only 6% of the samples predicted by Model 3 met the EMA criteria. In addition, the model failed to meet the defined thresholds for the predictive performance of both the MPPE and MAPE, indicating low reliability.

#### 2.2.3. Correction of the Ustekinumab Concentration in Capillary DBS Due to Irregular Spot Shape

**Figure 2:**
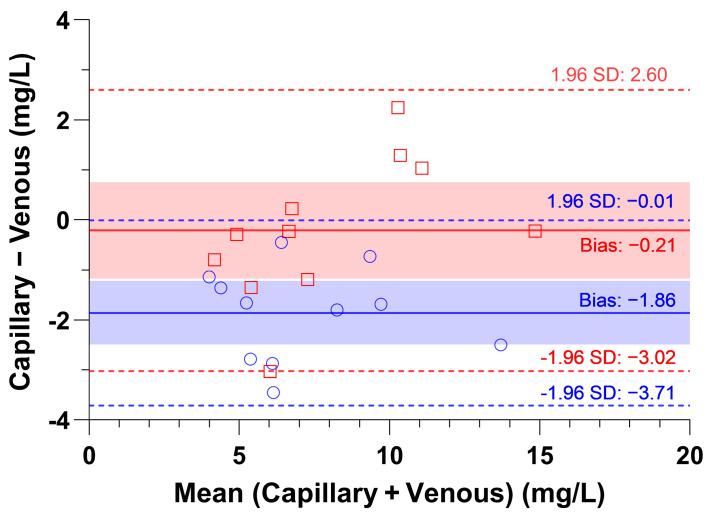
Bland–Altman analysis of UST concentrations in venous and capillary DBS. The capillary concentrations are shown in blue as uncorrected for the blood area on the DBS punch (circles) and in red as corrected concentrations (squares). The solid lines represent the absolute difference (bias), and the dashed lines indicate the limits of agreement. The values of the bias and limits of agreement are indicated. The shaded area represents the 95% confidence interval for bias.

Of the 25 capillary DBS samples obtained, 11 had irregular shapes that did not cover the entire area of the punch. To correct the measured UST concentrations in these samples, a correction strategy was applied using the DBS area of the punch. Figure 2 shows a Bland–Altman analysis comparing the UST concentrations in venous DBSs and the corrected or uncorrected UST concentrations in capillary DBSs. Analyses of uncorrected UST-DBS concentrations in capillary blood showed a systemic absolute bias and much wider LoA than those of UST-corrected concentrations in capillary DBSs. Our correction method was free of systemic bias (−0.21 mg/L; 95% CL: −1.18, +0.75) with narrow LoA (−3.02 to 2.60 mg/L). In addition, the correction strategy met the EMA criteria, as 81.8% of the samples were within 20% deviation from the mean. Finally, the corrected UST capillary DBS concentrations showed an MPPE of −3.35% and an MAPE of 13.3%, confirming comparable concentrations between venous and corrected capillary DBS concentrations.

#### 2.2.4. Correlation Between Ustekinumab Concentration in Venous and Capillary Dried Blood Spots

**Figure 3:**
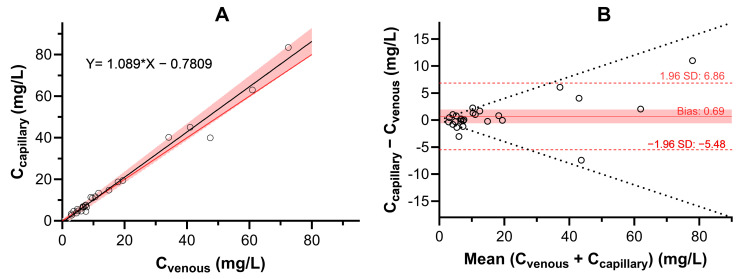
Deming regression (**A**) and Bland–Altman analysis (**B**) of UST concentrations in venous and capillary DBS samples. The DBS concentrations of UST in capillary blood were corrected for the blood area on the DBS punch. The Deming regression shows a linear regression line in a solid black line and a shaded red area indicating its 95% confidence interval. The red line represents the identity line. In the Bland–Altman plot, bias with 95% confidence interval is shown with solid red line and red area, respectively. The LoA are presented with dotted red lines, while the deviation for 20% of the mean concentrations is shown with a dotted black line.

To assess the correlation between the venous and capillary concentrations of UST in DBSs, 25 samples were compared using the dataset of capillary samples with corrected concentrations (Figure 3). The Deming regression showed that the slope was 1.09 with a 95% confidence interval of 0.94 to 1.24, and the intercept was −0.781 with a 95% confidence interval of −2.3 to 0.73. The capillary concentrations slightly overpredicted the venous concentrations; however, the difference was not significant. Moreover, Bland–Altman analysis confirmed the absence of systemic bias, since the average absolute bias was 0.69 mg/L, with a 95% confidence interval of −0.59 to 1.96 mg/L. The LoA were narrow (−5.5 to 6.9 mg/L), and 84% of the samples were within 20% deviation from the mean.

## 3. Discussion

Guthrie et al. [21] introduced the use of capillary blood for the detection of phenylketonuria in neonates in 1963. This novel method offers the advantage of fast and inexpensive screening of newborns. Since then, DBS has been implemented for TDM of various drugs, including mAbs. The present prospective cohort study applied the DBS method for TDM of UST in patients diagnosed with IBD. The developed method could be an important tool for dose adjustment in routine care, which also enables remote monitoring of patients, as it could allow sampling at home and subsequent UST measurements in specialized laboratories.

The commercial enzyme-linked immunosorbent assay (ELISA) method, developed for measuring UST concentrations in serum or plasma samples, underwent various optimization stages to enable measurement in DBS samples. First, different extraction conditions for sample preparation were thoroughly investigated. In our method, we opted for a 4 h extraction of DBSs. Prolonged extraction times of 8 and 12 h were also tested. The response ratio between 4 and 8 h was 1.01, indicating consistency between those extraction times. Similarly, the ratio between 4 and 12 h was 0.99, suggesting the response remained stable over the extended period. Therefore, we selected the extraction time which had the shortest turnaround and delivered accurate and precise results. Additionally, we evaluated different extraction solution compositions of Tween^®^ 20 (0.05% and 0.1% *v/v*) and a modified extraction solution containing bovine serum albumin (BSA; 0.1%, 0.5%, and 1% *v/v*). The objective of this alteration was to investigate whether BSA would act as a blocking agent to prevent non-specific binding of antibodies in the microtiter wells, but there was no significant effect on the results. An additional step in which extracts were centrifuged (5 min, 15,700× *g*) was also evaluated but offered no advantage to the method. Finally, we investigated the potential interference of materials (anticoagulants) from different types of collection tubes. We compared the blank responses of DBS samples obtained from blood collected in lithium heparin (Li-heparin) BD Vacutainers^®^ and BD Vacutainer^®^ serum tubes. The ratio of the response of blank DBS samples obtained from blood samples from the two types of tubes (Li-heparin/serum tube) was 0.99, indicating a minimal difference in the background signal between the two collection tubes. We also compared the responses of blank DBS samples obtained from blood collected in a Li-heparin tube and capillary blood directly deposited on a DBS card. In this case, the ratio of blank responses (Li-heparin blood/capillary blood) was 1.1, suggesting no significant matrix-related or anticoagulant interference.

The aim of the developed method was to measure UST concentrations in DBS samples collected during the early phase of treatment. Previous studies have shown that UST concentrations ≥4.2 mg/L at week 8 are associated with biochemical remission [22]. In addition, our previous studies have shown that UST concentrations as early as 1 h after the induction dose (≥104 mg/L) or after two weeks of therapy (≥27 mg/L) are associated with biochemical or endoscopic remission [9,23]. Therefore, the analytical method used for early drug optimization by TDM should cover these concentrations. The developed method range of UST in DBS samples was between 3 mg/L and 12 mg/L, with the upper limit of quantification (ULOQ) increasing to 120 mg/L when an additional dilution step was used. Based on the treatment week, we diluted the DBS extracts to obtain an optimal sample concentration. We opted for a 1:2000 dilution for DBS samples obtained at the beginning of treatment (week 0) and in the 2nd week of treatment. The DBS samples were then diluted 1:200. Our method can determine UST concentrations in DBS samples from patients from week 0 to week 12 and is therefore useful for early decision-making to optimize dosing. The validation process showed that the method met the validation parameters defined by the International Association for Therapeutic Drug Monitoring and Clinical Toxicology (IATDMCT) [20].

To confirm the validity of the developed DBS method, several DBS-specific validation parameters were analyzed. One of the most important parameters that can affect recovery, spot size, and blood-to-plasma concentration ratio is HCT [24]. The effect of HCT on selectivity and recovery was investigated following the previously mentioned guidelines [20]. The results of this study suggest that HCT influences selectivity; however, the investigated range of HCT was broad, ranging from 0.2 to 0.6. The typical HCT value of the IBD population is between 0.36 and 0.45 [14,25]. The HCT values of the patients included in the present study were in a similar range (0.35–0.46). A sub-analysis of the effect of HCT on selectivity confirmed that HCT in the range of 0.3–0.5 had no influence. The effect of HCT on recovery was analyzed using QC samples at high and low concentrations. The recovery was independent of the effect of HCT. Finally, the HCT effect was investigated according to the IATDMCT guidelines [20]. The deviations of UST concentrations from those of HCT 0.4 were less than 12%, confirming that HCT had no effect. Regarding the size of the spots, the narrow HCT range of the patients included in the study (0.35–0.46) is expected to contribute to minimal differences in the volume of blood collected after a 6 mm punch. The blood-to-plasma concentration ratio is an important parameter for evaluating PK properties. Hydrophilic compounds, therapeutic proteins (such as mAbs), and acidic drugs exhibit a high affinity for plasma proteins, restricting their partitioning into erythrocytes. Consequently, for these drugs, the blood-to-plasma concentration ratio approaches the lower boundary of 0.55–0.60. In such instances, blood cells primarily act as a diluent, and the primary concern, aside from the typical issue of altered plasma binding, is the HCT level, especially if the DBS analysis is utilized. In this study, the HCT remained relatively constant, indicating that the variation in value was minimal enough that it did not significantly impact the correlation between unbound plasma and total concentrations [26]. We extensively examined the stability of DBSs under different conditions for various time intervals. In addition to the prolonged periods at RT or −20 °C, we also tested the stability of UST on DBS samples under conditions that might be encountered while transporting the samples (especially during the summer period). We confirmed that a period of two days at 40 °C did not affect the UST stability of the DBS samples. In addition, the room conditions for one month had no influence on the UST stability of the DBS samples, which confirms the suitability of the microsamples for remote monitoring.

UST is a large molecule that is expected to be found exclusively in the plasma. To adequately interpret the UST DBS concentration for TDM purposes, we determined the relationship between serum and blood concentrations. A comparison of UST DBS with UST serum concentrations confirmed that the simple proportional and linear models adequately predicted serum concentrations. Because the linear model correctly predicted more than 71% of the samples, we suggest that it should be used to convert DBS concentrations to serum concentrations. However, no improvement in predictive performance was observed when HCT was introduced into the relationship between serum and DBS concentrations. This is consistent with the results of other studies that found that simple proportional or linear models adequately predicted the serum concentrations of golimumab and vedolizumab [12,18]. In contrast, studies on adalimumab and infliximab showed improved predictions when the model included fixed or individual HCT levels [14,16]. We believe that the lack of HCT effect on the predictive performance in our model is most likely due to the narrow HCT range observed in the patients studied. To support the developed method for remote TDM, we analyzed the correlation between UST concentrations in venous and capillary blood. The results demonstrated the absence of systemic bias, with a clinically insignificant overprediction of capillary concentrations compared to venous concentrations. However, this result was achieved only after an appropriate method to address the quality issues in DBS samples was developed. DBS samples obtained from venous blood were prepared volumetrically by spotting 20 μL of venous blood onto a paper card. The capillary DBS samples, on the other hand, were collected at the hospital center by the assistance of healthcare providers. Regardless of their assistance, only 56% of capillary samples adequately covered the punched area. By implementing image analysis, we were able to overcome the poor quality of the DBS samples and obtained concentrations that matched well with the venous samples. The developed method represents an important and novel tool for improving the quality of DBS samples [27,28].

A limitation of this study can be considered that capillary samples were collected with the assistance of healthcare professionals. This can positively affect the quality of the collected samples. Therefore, we propose a method to correct the DBS samples in cases where the spot exhibits an irregular shape. We acknowledge that the relatively small sample size in this study can be considered a limitation. Involving a bigger number of patients can be challenging in research involving TDM of mAbs, especially in our case, where a microsampling method was used. The primary aim was to investigate the feasibility and the clinical applicability of the method. Even with the relatively small number of patients, the paired venous and capillary samples (35 and 25, respectively) provided sufficient data to establish a correlation between serum and DBS samples. Moreover, patients diagnosed with IBD and receiving UST therapy represent a distinct and clinically important subgroup, verifying that the study had a targeted approach. Larger, multi-center studies are needed to confirm these results, improve the statistical power, and assess the application of the method across broader patient populations. We acknowledge that our study did not test for anti-drug antibodies (ADAs). We considered the low immunogenicity rates of UST during clinical trials [29,30] and the results of more recent studies [31]. The formation of ADAs can greatly influence the efficacy of therapy and is considered one of the main reasons for the failure of therapy with anti-tumor necrosis factor agents [32]. The reason for the low immunogenicity of UST remains unknown, but its different mechanism of action and the properties of the molecule itself might be responsible for it [31]. Nevertheless, future studies could integrate ADAs assessment for UST to further validate the robustness of DBS sampling in the presence of immunogenicity.

## 4. Materials and Methods

### 4.1. Chemical and Materials

DBS samples were collected on Whatman™ 903 Protein Saver Cards (GE Healthcare, Dassel, Germany). Drug-free venous blood was used to prepare the DBS calibrators and QC samples. It was obtained from healthy volunteers using Li-heparin BD Vacutainers^®^ (Becton and Dickinson, Franklin Lakes, NJ, USA). The ELISA kits and UST stock solution used to spike the blood were purchased from ImmunoGuide^®^ (AybayTech Biotechnology, Ankara, Turkey). A stainless-steel hole puncher from Rayher Hobby & Art (Laupheim, Germany) was used for the punches of the DBS samples. Ultrapure water obtained using an A10 Advantage Milli-Q water purification system (Millipore Corp., Billerica, MA, USA), sodium azide (Sigma-Aldrich, Steinheim, Germany), and Tween^®^ 20 (polyoxyethylene (20) sorbitan monolaurate; Merck, Darmstadt, Germany) was used to prepare the extraction solution.

### 4.2. DBS Method

In the first step, drug-free venous blood was collected in 6 mL Li-heparin BD vacutainers^®^ (Beckton Dickinson, Oxford, UK) from volunteer participants under the approved ethical guidelines. The HCT of the obtained blood was measured using a Microsemi CRP hematology analyzer (Kyoto, Japan). Subsequently, blood with HCT representative of the target population (patients diagnosed with IBD) was prepared using established procedures [33]. The HCT of the target population (0.4) was estimated based on the mean HCT value obtained from IBD patients. The spiked calibrators covered a predetermined range of UST concentrations (3, 4, 6, 9, and 12 mg/L). QC samples were prepared at three concentration levels: low (QC_L_ = 5 mg/L), medium (QC_M_ = 8 mg/L), and high (QC_H_ = 10 mg/L). To achieve these concentrations, a stock solution was used to spike venous blood, and 20 μL of spiked blood was spotted on the filter paper. The DBSs were air-dried for at least 3 h, avoiding direct sunlight. A punch of approximately 6 mm was taken from the central area of the DBSs and transferred into an Eppendorf tube with 500 μL of extraction solution prepared using phosphate-buffered saline containing 0.05% Tween^®^ 20 and 0.05% sodium azide (Merck, Darmstadt, Germany). The samples were then incubated for 4 h at 25 °C while being gently shaken at 200 rpm using the HeatMix Tehtnica (Domel, Slovenia). After incubation, the supernatant was collected, and UST concentrations were measured using an ELISA kit and a microplate reader (Tecan Safire; Tecan Group Ltd., Männedorf, Switzerland).

### 4.3. DBS Method Validation

Analytical validation was performed according to the IATDMCT guidelines [20]. The method was validated for selectivity, recovery, dilution integrity, linearity, accuracy, precision, lower and upper limits of quantification, and stability.

#### 4.3.1. Selectivity, Recovery, and Dilution Integrity

To evaluate selectivity, drug-free venous blood was obtained from five different donors, and HCT was measured. Blood was then used to prepare a wide range of HCT values (0.2, 0.3, 0.4, 0.5, and 0.6) [33]. The recovery of the developed method was determined at two QC concentration levels (QC_L_ and QC_H_) at two different HCT levels (0.25 and 0.55) in triplicate. Recovery was assessed by comparing the responses of pre-extraction spiked DBS samples (blood with known UST concentration was spotted on Whatman 903 cards, air-dried, and extracted) and post-extraction DBS samples (blank blood samples were spotted on Whatman 903 cards, air-dried, extracted, and the extracts were spiked with UST to match the concentration of pre-extraction samples). Dilution integrity was assessed by preparing two samples above the ULOQ, each in triplicate. The concentrations of these samples were 30 and 120 mg/L, and additional dilution steps of 5-fold and 10-fold were added, respectively. The acceptance criterion was a deviation of <15%.

#### 4.3.2. Calibration Model, Accuracy, Precision, and Limits of Quantification

The calibrators were prepared in duplicate using freshly obtained venous blood for 5 days, and the HCT was adjusted to a value representative of the target group (0.4). Least-squares linear regression was used to determine the calibration curve based on the response of the calibrators and their nominal concentrations. The back-calculated concentrations of the calibrators should be within ±15% of the nominal values, and the LLOQ should be ±20%. The intra- and inter-day accuracy and precision were determined at four QC levels (LLOQ, QC_L_, QC_M_, and QC_H_) in whole blood obtained from donors with an adjusted HCT of 0.4. The QCs were analyzed in quintuplicate on five different days. The accuracy was obtained by comparing the QC concentration with the nominal value, and the precision was determined as the relative standard deviation (RSD).

#### 4.3.3. Hematocrit Effect

To assess the HCT effect, DBS-QC samples at low and high levels were prepared in triplicate using blood with HCT values of 0.25, 0.4, and 0.55. UST concentrations should be within ±15% of the concentration at the target HCT (0.4).

#### 4.3.4. Stability

The stability of UST on DBS cards was assessed by spiking different concentrations of UST at two QC levels (QC_L_ and QC_H_) in triplicate using blood with an adjusted HCT of 0.4 and spotting the filter paper cards. The cards were air-dried for at least 3 h and subsequently stored in an airtight bag with a desiccant, avoiding direct sunlight, either at RT or at −20 °C. The UST concentration was measured and compared with that of the freshly prepared samples. The stability of UST was also investigated under conditions that might be met during the transportation of the samples to the respective laboratories for further analysis, especially during summer; therefore, they were placed in an oven for 48 h at 40 °C. The effect of storage conditions on the UST samples was determined with an absolute mean percentage deviation ≤15% as the minimum acceptance criteria.

### 4.4. Patients’ Samples

Adult patients diagnosed with IBD, and healthy volunteers were recruited for the prospective observational study, which was approved by the National Medical Ethics Committee of the Republic of Slovenia (0120-013/2016-2, KME 38/01/16) and was conducted according to the Declaration of Helsinki. Blood samples included in this study were obtained from patients who started their treatment with UST during the programmed outpatient visit at the Department of Gastroenterology, University Medical Centre Ljubljana. They received the UST intravenous induction dose based on body weight (≤55 kg, 260 mg; >55 to ≤85 kg, 390 mg; >85 kg, 520 mg), followed by a subcutaneous 90 mg dose every 8 or 4 weeks. Blood samples were obtained by venipuncture at baseline, 1 h after the induction dose, and subsequently at weeks 2, 4, 8, 10, 12, and 16 [23]. Paired DBS and serum samples were then prepared. DBS samples were prepared as described above, while serum samples were prepared by blood collection in Vacutainer serum tubes (Becton and Dickinson, Franklin Lakes, US), centrifugation, and storage at −80 °C until further analysis. Some patients agreed to simultaneous collection of venous and capillary blood. Capillary samples were obtained by finger pricking, followed by the preparation of DBS capillary samples with the assistance of healthcare professionals. Serum samples were analyzed using an ELISA kit according to the manufacturer’s instructions.

### 4.5. Clinical Validation

Clinical validation was performed using paired patient samples to determine the correlation between UST serum and venous DBS concentrations, as well as between venous DBS (DBSv) and capillary DBS (DBSc) concentrations. Three different strategies were used to determine the correlations between serum and DBS concentrations: (i) the proportional model predicted serum concentration based on individual DBSv concertation and constant *k^min^* obtained by the nonlinear regression between the experimental data and model predictions (Equation (1)); (ii) the linear model predicted serum concentration based on individual DBSv concentration, slope (1/*k^min^*), and intercept (*b^min^*) obtained by nonlinear regression (Equation (2)); and (iii) the last model predicted serum concentration based on individual DBSv concentration and individual HCT.

(1) Model 1: Csi=Cdbs vikmin 

(2) $$Model\;2:\;C_{s}^{i}=\frac{C_{dbs\;v}^{i}}{k^{min}}+b^{min}$$

(3) $$Model\;3:\;C_{s}^{i}=\frac{C_{dbs\;v}^{i}}{\left(1-{Hct}^{i}\right)}$$

The relationship between the measured UST serum concentration and the model-predicted concentration was assessed using Deming regression and Bland–Altman analysis. The acceptance criteria were differences within 20% of the mean for at least 67% of the samples [34]. Additionally, the predictive performance was assessed by calculating MPPE and MAPE. MPPE and MAPE values <15% were considered acceptable.

**Figure 4:**
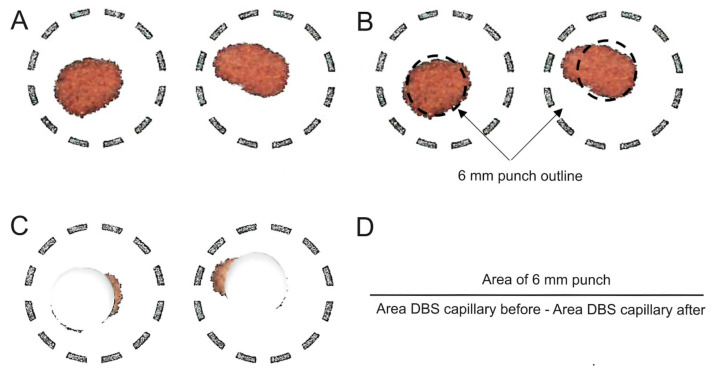
Analysis of DBS capillary samples in cases where the DBS had an irregular shape or the area of the 6 mm punch was not completely filled with capillary blood. (**A**) DBS scans showing the total area of the DBS sample with an irregular shape. (**B**) DBS scans showing the marked area of the 6 mm DBS punch before the punching process. (**C**) DBS scans showing the area of the DBS after the punching process. (**D**) Equation for calculating the correction factor.

The relationship between UST concentrations in venous and capillary DBS samples was determined using Deming regression and Bland–Altman analysis. If the capillary DBS sample had an irregular shape that affected the area of the 6 mm punch, which had to be completely covered with blood and thus affected the volume of capillary blood analyzed, the DBS card was scanned using a simple flatbed scanner before and after the punch. The scans were analyzed using ImageJ^®^ software (version 1.54p) to estimate the area of the DBS sample. The 6 mm punch area (a constant area as all experiments were performed with the same punch) was divided by the difference in the area of the capillary DBS before and after the punch to obtain the factor used to correct for the volume of blood analyzed in the punch of the capillary DBS sample (Figure 4).

### 4.6. Data Analysis

All data analyses were performed using Microsoft Excel 2019 MSO, GraphPad Prism 10, and IBM SPSS Statistics for Windows version 29. For multiple comparisons, one-way ANOVA with Bonferroni post hoc test was used. Statistical significance was set at *p* < 0.05.

## 5. Conclusions

The current study presents development, validation, and clinical application of a novel DBS method for the TDM of UST in patients with IBD. This is a patient-centric sampling method that offers advantages over venipuncture. Early estimation of serum UST levels could help predict which patients are more likely to achieve remission. The newly developed DBS method is the first to enable reliable measurement of UST in capillary blood, monitoring of patients, and appropriate clinical interpretation of the measured UST concentrations. This approach could allow for proactive TDM by optimizing the dose in patients who are unlikely to achieve remission with standard dosing; however, further multi-center studies with larger cohorts are needed to confirm clinical applicability of the developed method.

## Figures and Tables

**Table 1:** Linear response range, intercept values, slope, and coefficient of determination (r^2^) for the calibration curves of UST in DBS calibrators.

Linear Response Range (mg/L)		Intercept	Slope	r^2^
Day 1	3–12 (mg/L)	0.2011	0.0955	0.9858
Day 2		0.3188	0.0969	0.9984
Day 3		0.1527	0.0892	0.9912

r^2^—coefficient of determination.

**Table 2:** Intra- and inter-day precision and accuracy for UST in samples at LLOQ and QC levels.

Intra-Day Precision					Intra-Day Accuracy		
*n* = 5	Level	Nominal (mg/L)	Mean (mg/L)	RSD (%)	*n* = 5	Level	%
	LLOQ	3.0	2.7	6.6		LLOQ	88.5
	QC_L_	5.0	5.1	9.1		QC_L_	103
	QC_M_	8.0	6.7	13.4		QC_M_	84.2
	QC_H_	10.0	11.1	1.2		QC_H_	111
**Inter-Day Precision**					**Inter-Day Accuracy**		
*n* = 15	Level	Nominal (mg/L)	Mean (mg/L)	RSD (%)	*n* = 15	Level	%
	LLOQ	3.0	2.7	6.3		LLOQ	90.1
	QC_L_	5.0	5.3	11.2		QC_L_	106
	QC_M_	8.0	8.1	6.3		QC_M_	102
	QC_H_	10.0	9.4	6.4		QC_H_	94.4

*n*—number of samples; RSD—relative standard deviation; LLOQ—lower limit of quantification; QC_L,M,H_—quality control samples at low, medium, and high concentrations.

**Table 3:** Hematocrit effect on UST concentration in DBS.

QC	HCT	Nominal (mg/L)	Mean (mg/L)	RSD (%)	C_HCTi_/C_HCT 0.4_ (%)
QC_L_	0.25	5.0	5.8	3.59	112
QC_L_	0.4	5.0	5.1	9.14	100
QC_L_	0.55	5.0	5.5	6.48	107
QC_H_	0.25	10.0	12.2	5.72	107
QC_H_	0.4	10.0	11.1	1.23	100
QC_H_	0.55	10.0	11.6	1.18	105

HCT, hematocrit; RSD, relative standard deviation; C_HCTi_/C_HCT 0.4_, ratio of the UST concentration in DBS obtained with specific blood HCT and UST concentration in DBS obtained with blood with 0.4 HCT; QC_L,M,H_, quality control samples at low, medium, and high concentrations.

**Table 4:** Patient characteristics.

**Age ^a^**	[years], median (Q1–Q3)	52.4 (42.0–57.9)
**Sex**	(nM/nW)	5/5
**BW ^a^**	[kg], median (Q1–Q3)	76.5 (67.5–92.5)
**CRP ^a,b^**	[mg/L], median (Q1–Q3)	9.5 (6.5–12.5)
**HCT**	[], median (Q1–Q3)	0.40 (0.39–0.44)
**FC ^a,c^**	[mg/kg], median (Q1–Q3)	130 (44.5–215)
**S-alb ^a^**	[g/L], median (Q1–Q3)	43.0 (41.0–46.0)
**Dose initial ^a^**	[mg], median (Q1–Q3)	390 (390–520)
**Dose maintenance ^d^**	[mg/week], (number of patients)	90 mg/week 8, (9) 90 mg/week 4, (1)
**Week of therapy ^d,e^**	[week], median (Q1–Q3)	4.0 (2.0–8.6)

BW—body weight; CRP—C-reactive protein; HCT—hematocrit; FC—fecal calprotectin; S-alb—serum albumin concentration; n_M_—number of men; n_W_—number women; Q1—first quartile (25th percentile); Q3—third quartile (75th percentile); ^a^ initiation therapy; ^b^ reported values are for 4 patients, as 6 patients had values below 5 mg/L; ^c^ reported values are for 3 patients, as 3 patients had values over 500 mg/kg, and for one patient, the data are missing; ^d^ maintenance therapy; ^e^ week of therapy at UST concentration measurement

**Table 5:** Agreement and predictive performance of the models for estimating UST concentration in serum from measured concentration in DBS venous samples.

Predicting Model		Deming Regression		Bland–Altman Analysis		Predictive Performance	
		Slope (95% CI)	Intercept (95% CI)	Bias (mg/L) (95% CI)	95% LoA (mg/L)	MPPE (%)	MAPE (%)
1	$C_{s}^{i}=\frac{C_{dbsv}^{i}}{k^{min}}$ *k^min^* = 1.15	0.999 (0.84, 1.16)	−0.541 (−3.38, 2.30)	−0.554 (−2.45, 1.35)	−11.04, 9.94	−2.19	14.44
2	$C_{s}^{i}=\frac{C_{dbs\;v}^{i}}{k^{min}}+b^{min}$ *k^min^* = 1.17; *b^min^* = 1.04	0.979 (0.82, 1.13)	0.523 (−2.26, 3.31)	−1.71E−5 (−1.83, 1.83)	−10.4, 10.4	2.94	15.54
3	$C_{s}^{i}=\frac{C_{dbs\;v}^{i}}{\left(1-{HCT}^{i}\right)}$	2.016 (1.54, 2.49)	−2.646 (−10.6, 5.34)	22.85 (13.4, 32.3)	−31.1, 76.8	83.74	83.74

LoA—limit of agreement; 95% CI—95 confidence interval; MPPE—median predictive percent error; MAPE—median absolute predictive error; $C_{s}^{i}$—individual serum concentration; $C_{dbsv}^{i}$—individual DBS venous concentration; model 1, *k^min^*—constant obtained by the nonlinear regression; model 2, slope (*k^min^*) and intercept (*b^min^*) both obtained by nonlinear regression; ${HCT}^{i}$—individual hematocrit.

## Data Availability

The data presented in this study are available upon reasonable request from the corresponding author.

## Supplementary Materials

The following supporting information can be downloaded at: https://www.mdpi.com/article/10.3390/ph18091253/s1, Table S1: ELISA responses of blank DBS samples prepared from five donors with adjusted HCT. Table S2. Accuracy and precision of samples used for dilution integrity analysis.

## Abbreviations

The following abbreviations are used in this manuscript:

95% CI	95% Confidence Interval
ADAs	Anti-drug antibodies
*b^min^*	Intercept obtained by nonlinear regression
BSA	Bovine serum albumin
BW	Body weight
CD	Crohn’s disease
C_HCT 0.4_	UST concentration in DBS obtained with 0.4 hematocrit
C_HCTi_	UST concentration in DBS obtained with specific blood hematocrit
CRP	C-reactive protein
DBS	Dried blood spots
dbs c	Uncorrected dried blood spot area
dbs c corr	Corrected dried blood spot area
DBSc	Capillary dried blood spots
DBSv	Venous dried blood spots
ELISA	Enzyme-Linked Immunosorbent Assay
EMA	European Medicines Agency
FC	Fecal calprotectin
HCT	Hematocrit
HCTi	Individual hematocrit
IATDMCT	International association for Therapeutic Drug Monitoring and Clinical Toxicology
IBD	Inflammatory bowel disease
IL	Interleukin
*k_min_*	Slope obtained by nonlinear regression
Li-heparin	Lithium heparin
LLOQ	Lower Limit of Quantification
LoA	Limits of Agreement
mAb	Monoclonal antibody
MAPE	Mean absolute percentage error
MPPE	Mean predictive percent error
n_M_	Number of men
n_W_	Number of women
PK	Pharmacokinetics
Q1	First quartile (25th percentile)
Q3	Third quartile (75th percentile)
QC_H_	Quality control high concentration
QC_L_	Quality control low concentration
QC_M_	Quality control medium concentration
RSD	Relative standard deviation
RT	Room temperature
S-alb	Serum concentration of albumin
TDM	Therapeutic drug monitoring
UC	Ulcerative colitis
ULOQ	Upper limit of quantification
UST	Ustekinumab
