# Supplementary material for: Development, Validation and Application of the Dried Blood Spot Analysis Method for the Determination of Ustekinumab in Patients with Inflammatory Bowel Disease
Source: Pharmaceuticals (Basel). 2025 Aug 24;18(9):1253. doi: 10.3390/ph18091253 (PMC12472599; doi:10.3390/ph18091253)
Supplement: Supplementary file 1 [file pharmaceuticals-18-01253-s001.zip › pharmaceuticals-3815275-supplementary.pdf]

Supplementary material:

Table S1. ELISA responses of blank DBS samples prepared from 5 donors with adjusted HCT.

Subject	HCT 0.2	HCT 0.3	HCT 0.4	HCT 0.5	HCT 0.6
1	0.209	0.187	0.189	0.152	0.136
2	0.296	0.261	0.277	0.229	0.194
3	0.324	0.231	0.204	0.209	0.194
4	0.285	0.232	0.211	0.187	0.168
5	0.191	0.195	0.183	0.167	0.166
Average	0.261	0.221	0.213	0.189	0.172
SD	0.058	0.030	0.038	0.031	0.024
RSD	22.2	13.7	17.7	16.5	14.1

HCT – hematocrite, SD – standard deviation, RSD – relative standard deviation

ANOVA analysis showed that the ELISA responses of blanks for hematocrites ranging from 0.2 to 0.6 were significantly different (p=0.015, $F_{df1=4, df2=20}$ = 4.031). Post hoc Bonferroni test showed significant difference between responses of samples with HCT 0.2 and 0.6 (p=0.013). As the population studied had an HCT value between 0.3 and 0.5 we performed ANOVA analysis for samples with HCT ranging from 0.3 to 0.5. No significant difference was observed (p=0.311, $F_{df1=2, df2=12}$ = 1.288), therefore, no corrections for HCT were made to the responses of the DBS samples obtained from patients.

Table S2. Accuracy and precision of samples used for dilution integrity analysis.

Nominal (mg/L)	Mean (mg/L)	Accuracy (%)	Precision (%)
30.0	28.8	96.0	8.27
120	113.4	94.5	7.74

n=3
